# MiR-652-5p elevated glycolysis level by targeting TIGAR in T-cell acute lymphoblastic leukemia

**DOI:** 10.1038/s41419-022-04600-7

**Published:** 2022-02-14

**Authors:** Shan Liu, Haobiao Wang, Wei Guo, Xiaoyan Zhou, Yi Shu, Haiyan Liu, Li Yang, Shi Tang, Hongyu Su, Ziyang Liu, Lamei Zeng, Lin Zou

**Affiliations:** 1 Center for Clinical Molecular Laboratory Medicine/Newborn Screening Center of Children’s Hospital of Chongqing Medical University, Chongqing, 400014 China; 2 National Clinical Research Center for Child Health and Disorders (Chongqing), Chongqing, 400014 China; 3 Ministry of Education Key Laboratory of Child Development and Disorders, Chongqing, 400014 China; 4 China International Science and Technology Cooperation Base of Child Development and Critical Disorders, Chongqing, 400014 China; 5 Center for Clinical Laboratory of Clinical Medical College & Affiliated Hospital of Chengdu University, Chengdu, Sichuan 610081 China; 6 Center for Clinical Laboratory of the Affiliated Hospital of Qingdao University, Qingdao, Shandong 266101 China; 7 Center for Pediatric Hematology Diseases of Children’s Hospital of Chongqing Medical University, Chongqing, 400014 China; 8 Center for Hematology Diseases of First Affiliated Hospital of Chongqing Medical University, Chongqing, 400014 China; 9 Clinical Research Unit, Children’s Hospital of Shanghai Jiao Tong University, Shanghai, 200062 China; 10 Institute of Pediatric Infection, Immunity, and Critical Care Medicine, Shanghai Jiao Tong University School of Medicine, Shanghai, 200062 China

**Keywords:** Paediatric cancer, Mechanisms of disease

## Abstract

The effect of glycolysis remains largely elusive in acute T lymphoblastic leukemia (T-ALL). Increasing evidence has indicated that the dysregulation of miRNAs is involved in glycolysis, by targeting the genes coding glycolysis rate-limiting enzymes. In our previous studies, we found that overexpression of the ARRB1-derived miR-223 sponge repressed T-ALL progress and reduced the expression of miR-652-5p. However, little is known about miR-652-5p on T-ALL. Here, we showed that impaired miR-652-5p expression inhibited growth, promoted apoptosis of T-ALL cells in vitro and prolonged overall survival (OS) in vivo. Based on the GO enrichment of miR-652-5p target genes, we uncovered that impaired miR-652-5p decreased glycolysis, including reduced the lactate, pyruvate, ATP level and the total extracellular acidification rate (ECAR), elevated oxygen consumption rate (OCR) in T-ALL cell lines. Mechanically, miR-652-5p targeted the 3ʹUTR of Tigar mRNA and inhibited its expression. Furthermore, the alteration of glycosis level was attributed to Tigar overexpression, consistent with the effect of impaired miR-652-5p. Additionally, Tigar suppressed the expression of PFKFB3, a glycolysis rate-limiting enzyme, in vivo and in vitro. Taken together, our results demonstrate that impaired miR-652-5p/Tigar axis could repress glycolysis, thus to slow growth of T-ALL cells, which support miR-652-5p as a novel potential drug target for T-ALL therapeutics.

## Introduction

T-cell acute lymphoblastic leukemia (T-ALL) was recognized as an independent disease in the 1970s, after discovering thymus-associated markers expressed on the surface of leukemic cells from pediatric patients [1]. Pediatric T-ALL has unfavorable features including high leukocyte count, hematopoietic failure, and medullar and extramedullar infiltration. The overall cure rates have increased to 80% in recent years due to improved diagnostic technologies and new treatment regimens [2]. However, the 5-year event-free survival rate of T-ALL is still significantly lower than that of other acute lymphoblastic leukemias [3]. Thus, novel and efficient therapeutics for T-ALL need to be urgently developed based on the new mechanism of leukemogenesis.

Aerobic glycolysis, which is defined as the uptake of glucose and secretion of lactate even when oxygen is present, is involved in the progression of cancers [4]. Moreover, the development of leukemia relies on high levels of glycolysis which may be a target for therapeutic intervention in acute myeloid leukemia [5, 6]. The increased glycolysis level is also found in T-ALL [7–9], and is elevated by oncogenes such as NOTCH [7] and RUNX2 [9]. However, the efficacy of glycolysis is reversed [7] or weakened [9] in T-ALL, implying that the mechanisms of glycolysis in T-ALL need to be explored.

MicroRNAs (miRs) are a group of small, noncoding RNAs that negatively regulate gene expression by the translational repression of target mRNAs [10]. Endogenous miRs play important roles in a variety of physiological processes including T-cell development [11]. Dysregulated miRs as oncomiRs [12, 13] or suppressors [14, 15] initiate and develop T-ALL. Our group previously reported that miR-223, as an oncomiR, suppressed NOTCH1 degradation by targeting ARRB1, thus promoting T-ALL cell proliferation [13]. Recently, we also demonstrated that impaired ARRB1 promoted the expression of miR-652-5p in T-ALL cell lines (accepted by Zhongguo Shi Yan Xue Ye Xue Za Zhi in May 2021). However, little is known about the role of miR-652-5p in T-ALL.

Given that miRs are reported to affect glycolysis level by targeting the genes coding glycolysis rate-limiting enzymes, such as PFKFB3 [16], GLUT-1 [17], and PFK1 [18], we reported that impaired miR-652-5p could slow the cell growth of T-ALL in vitro and prolong the survival time in vivo. We then found that impaired miR-652-5p could inhibit glycolysis by targeting TIGAR, thus suppressing PFKFB3 expression, which explained the molecular mechanism of T-ALL.

## Materials and methods

### Materials

All cell lines were preserved and identified in our laboratory by Shu et al. [13]. All cell lines were cultured according to the manufacturer’s instructions and confirmed as Mycoplasma negative by PCR methods, cellular experiments were performed within 20 passages after thawing. Anti-Tigar antibody(Cat No:22136-1-AP), anti-PFKFB3 antibody(Cat No:13763-1-AP) and anti-GAPDH antibody(Cat No:60004-1-lg) were obtained from proteintech (Rosemont, USA), and were used after dilution (1:1000, 1:1000 and 1:10000 respectively).

### Patient samples and isolated mononuclear cells

The enrollment and human subject protection plans for patients with T-ALL involved in this study were approved by the ethics committee of Chongqing Medical University Affiliated Children’s Hospital, Chongqing, China. Prior to the collection and use of the clinical samples, patients and their guardians were provided with detailed information about the benefits and risks of the study. The written informed consent forms were signed by the guardians during hospitalization at the Children’s Hospital of Chongqing Medical University according to the Declaration of Helsinki. The completed consent forms were placed in files. All essential patient identifiers were removed to protect the patients’ privacy.

Bone marrow specimens from patients with T-ALL or thymus tissue from healthy children were diluted with cold phosphate-buffered saline. The diluted cells were isolated for mononuclear cells by the Ficoll–Paque method (Cytiva, USA).

### Cell viability

Cells were firstly counted, and approximately 5 × 10^4^ cells per well were seeded in a 96-well cell culture plate. Then, after incubation at 37 °C in a humidified atmosphere with 5% CO_2_ for 48 h, 10 μL of the CCK-8 reagent (Dojindo, Shanghai, China) was added into each well, and absorbances (Abs) at 450 nm was measured using a multifunction microplate reader (BioTek, Vermont, USA) after incubation for 2 h at 37 °C. In the blank group, the well was only contained culture medium. Herein, the cells viability = (Abs of experimental group-Abs of blank group)/(Abs of control group-Abs of blank group) x 100%.

### Cell apoptosis

5 × 10^5^cells were collected and washed twice by cold PBS. The apoptosis was determined by Annexin V-APC/7-AAD apoptosis detection kit (keyGEN BioTECH, Taiwan, China), and analyzed by Flow cytometry (BD, USA).

### Detection of intracellular reactive oxygen species

A fluorescent probe (H_2_DCFDA) was used to detect the intracellular levels of reactive oxygen species (ROS) (Keygen Biotech, Jiangsu, China). Diluted H_2_DCFDA (1:1) was added, and the cells were incubated at 37 °C for 30 min. After incubation, the cells were washed with a serum-free medium to remove the external H_2_DCFDA three times. The fluorescence was monitored using a fluorescence microscope (Olympus, Japan) with excitation wavelength at 488 nm and emission wavelength at 530 nm.

### Measurements of intracellular ATP levels

An ATP Assay Kit (Beyotime, Shanghai, China) was used to detect the intracellular levels of ATP following the manufacturer’s protocols. About 5 × 10^5^ cells were resolved with ATP lysis buffer at 0 °C; the working solution was added; and the absorbance was measured using a microplate reader (GloMax 20/20 Luminometer). The absorbance value was used to calculate the ATP level by comparison with a standard curve.

### Lactate and pyruvate assay

The supernatant was collected from cell culture; MGA (Sigma, USA) was added as an internal standard (IS) for quantitative analysis; and the organic layer was carefully isolated after treatment with strong acid, strong alkali, and ethyl acetate. The levels of lactate and pyruvate were detected using gas chromatography–mass spectrometry (GC-MS) (Shimadzu, Japan), and the concentrations were quantified to IS.

### Extracellular acidification rate assay (ECAR)

The cells were pretreated with CO_2_ free for 3 h and planted in 96-well plates (5 × 10^5^ cells/well). The solution, oligomycin, and glycolytic inhibitor 2-DG were injected into each well following the manufacturer’s protocols (Abcam, USA). A fluorescence microplate analyzer was used to detect the fluorescence value every 1.5 min during 1.5 h at 380 nm excitation and 615 nm emission.

### Oxygen consumption rate assay

An Oxygen Consumption Rate (OCR) Assay Kit (Abcam, USA) was used for the real-time kinetic analysis of extracellular oxygen consumption rates following the manufacturer’s protocols. The cells were planted in 96-well plates (2 × 10^6^ cells/well). The solution and mineral oil were added to each well. The fluorescence microplate analyzer was used to detect every 1.5 min during 1.5 h at 380 nm excitation and 650 nm emission.

### Establishment and assessment of mice with leukemia

The use and care of the experimental animals was approved by the Ethics Committee of Chongqing Medical University. NOD/SCID mice (male, 6-8 week-year old) were randomly divided into three groups and were exposed to γ-ray radiation at doses of 2.3 Gy/min. Then, acute 5 × 10^5^ T leukemic cells marked as GFP- and firefly luciferase-tagged were injected into NSG mice via the tail vein. The weight every day and the in vivo imaging of leukemic cells every 5 days were monitored, and the survival time was recorded. The mice were sacrificed 50 days after injecting the leukemic cells. Spleen, liver, and bone marrow tissues of all mice were collected.

### Luciferase reporter assay

First, reporter plasmids were constructed with insert sequences (detailed sequence listed in Supplementary Table 2). Then, the cells were incubated in 96-well plates (1 × 10^4^ cells/plate) and transfected with the constructed reporter plasmids (10 μg) and pRL-SV40 (0.01 μg) according to the handbook of effect transfection reagent (Qiagen, Germany). Luciferase activity was measured with a Dual-Glo Luciferase Assay System (Promega, USA) and normalized against the Renilla luciferase activity. All experiments were performed at least three times in each plasmid and represented as the average relative luciferase activity.

### Statistical analysis

All data were done at least three independent experiments. Data were analyzed using two-sided *t* student among tested groups. For all statistical analyses, GraphPad Prism 5.0 software was used with a significance of *p* value < 0.05.

## Results

We analyzed and identified the expression of miR-652-5p first to demonstrate the possible role of miR-652-5p as a risk factor in T-ALL. Besides “oncomiR star”: miR-223-3p, miR-191-3p, miR-15a-3p, and so forth, increased miR-652-5p level was also observed in RNA-Seq data [13] (Supplementary Fig. 1A) and in GEO database GSE89978 (Supplementary Fig. 1B), compared with the healthy controls. Further, the average expression level of miR-652-5p was significantly upregulated in bone marrow specimens from patients with T-ALL (*n* = 13), compared with healthy controls (*n* = 5) (*p* = 0.04) (Supplementary Fig. 1C and Supplementary Table 1). And, the expression of miR-652-5p was diverse in T-ALL cell lines; increased expression was observed in Jurkat and Molt-4 cells (Supplementary Fig. 1D). These data implied that the expression of miR-652-5p positively correlated with T-ALL cells.

**Fig. 1:**
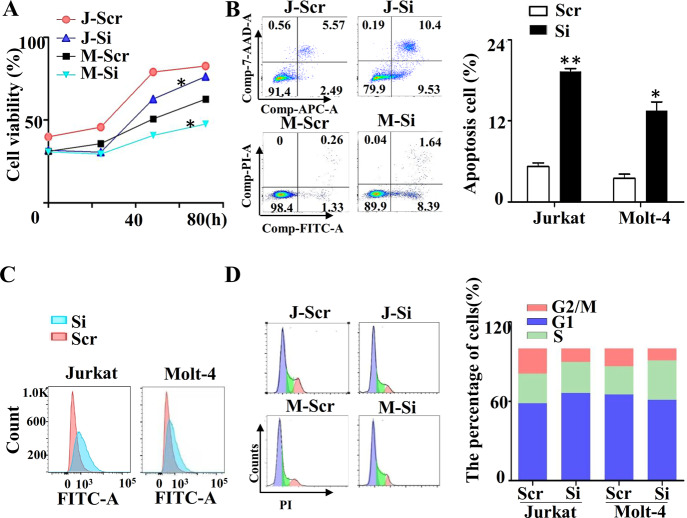
The role of miR-652-5p in T-ALL cells. Effect of impaired miR-652-5p on (**A**) growth by CCK-8 assay, (**B**) apoptosis by Flow cytometry, (**C**) ROS by Flow cytometry, and (**D**) cell cycle by Flow cytometry. *Abbreviation*: h, hours; J, Jurkat cell lines; M, Molt-4 cell lines; Scr, control; Si, impaired miR-652-5p. *p* is compared to control and calculated by *t* student; *p < 0.05; **p < 0.01.

To determine the role of miR-652-5p in T-ALL, we first constructed the stable cell lines with impaired miR-652-5p expression(Supplementary Fig. 2A) and identified the expression the expression of miR-652-5p(Supplementary Fig. 2B). We found that the cell growth rate of Jurkat and Molt-4 cells reduced dramatically via impaired miR-652-5p expression (named J-Si and M-Si, respectively) (Fig. 1A), compared with control cells (named J-Scr and M-Scr, respectively), but the growth rates of Loucy and P12 cells were not different (Supplementary Fig. 2C). Therefore, Jurkat and Molt-4 cells were used for further investigation. The effects of miR-652-5p on apoptosis, ROS levels, and cell cycle were analyzed by flow cytometry. The impaired miR-652-5p expression significantly promoted the apoptosis of T-ALL cells (Fig. 1B) and slightly increased the ROS level (Fig. 1C). J-Si was arrested in the G1 stage of the cell cycle, but M-Si was arrested in the S stage (Fig. 1D). These results suggested that impaired miR-652-5p expression might inhibit the growth of T-ALL cells via multiple biological functions of cells.

**Fig. 2:**
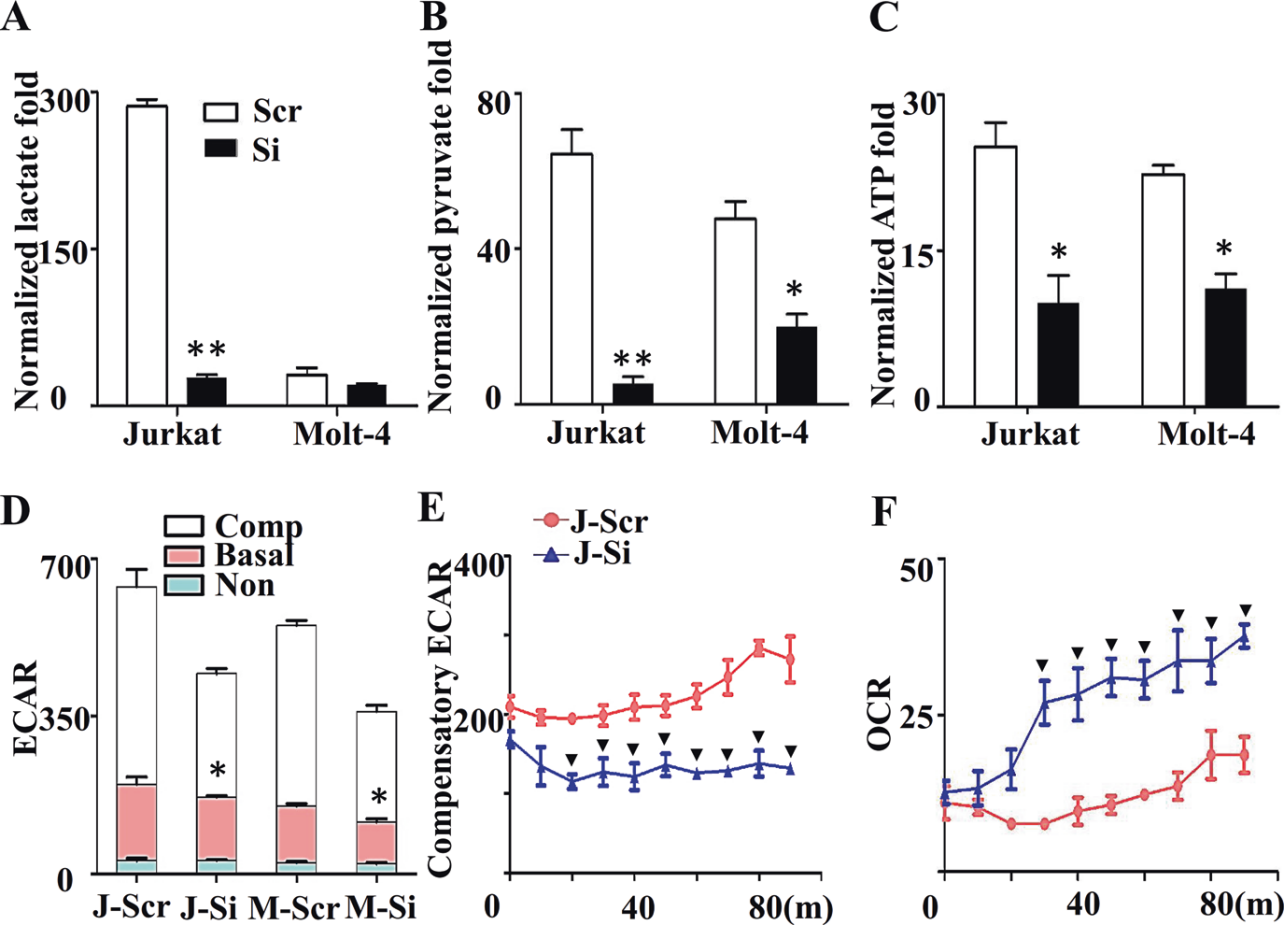
The role of miR-652-5p in glycolysis of T-ALL cells. Gas Chromatography-Tandem Mass detected (**A**) the lactate content, (**B**) pyruvate content, and the value was normalized to internal control. (**C**) Effect of impaired miR-652-5p on the production of ATP. (**D**) Extracellular acidification rate (ECAR) at end point, (**E**) Continuous monitoring compensatory ECAR, and (**F**) Oxygen consumption rate (OCR) were detected by fluorescence microplate analyzer. m minutes, J Jurkat cell lines, M Molt-4 cell lines, Scr control, Si impaired miR-652-5p, ECAR extracellular acidification rate, OCR oxygen consumption rate. Non, the ECAR from non-glycosis; Comp, compensatory glycosis from stimulation with oligo. *p* is compared to control and calculated by *t* student; *p < 0.05; **p < 0.01., indicated the significance at certain time point, compared to control, *p* < 0.05.

To clarify the possible mechanism, we predicted the target genes of miR-652-5p using miRbase software and TargetScan 7.0 software, and obtained 205 possible target genes by the intersection analysis. Based on the Gene Ontology (GO) term enrichment using DAVID software, the critical role of glucose metabolism (GO: 0009749 and GO: 0006003) was determined, including PFKFB2 and TIGAR (Supplementary Fig. 3A). Previous studies reported that PFKFB2 [19] and TIGAR [20] were involved in glycolysis. Therefore, we detected the effect of miR-652-5p on the glycolysis level in T-ALL cells. GC-MS was carried out to evaluate glycolysis. We found that impaired miR-652-5p expression significantly decreased the lactate and pyruvate levels (Figs. 2A and 2B). The ATP assay indicated that impaired miR-652-5p expression reduced the ATP level (Fig. 2C). The extracellular acidification rate and the OCR in J-Si and M-Si cells were determined using the XF metabolic flux analyzer. The ECAR markedly decreased in J-Si and M-Si cells, especially compensatory glycolysis (Figs. 2D and 2E and Supplementary Fig. 4A), and more oxygen was consumed (Fig. 2F and Supplementary Fig. 4D). These results confirmed that impaired miR-652-5p expression dampened glycolysis in T-ALL cells.

**Fig. 3:**
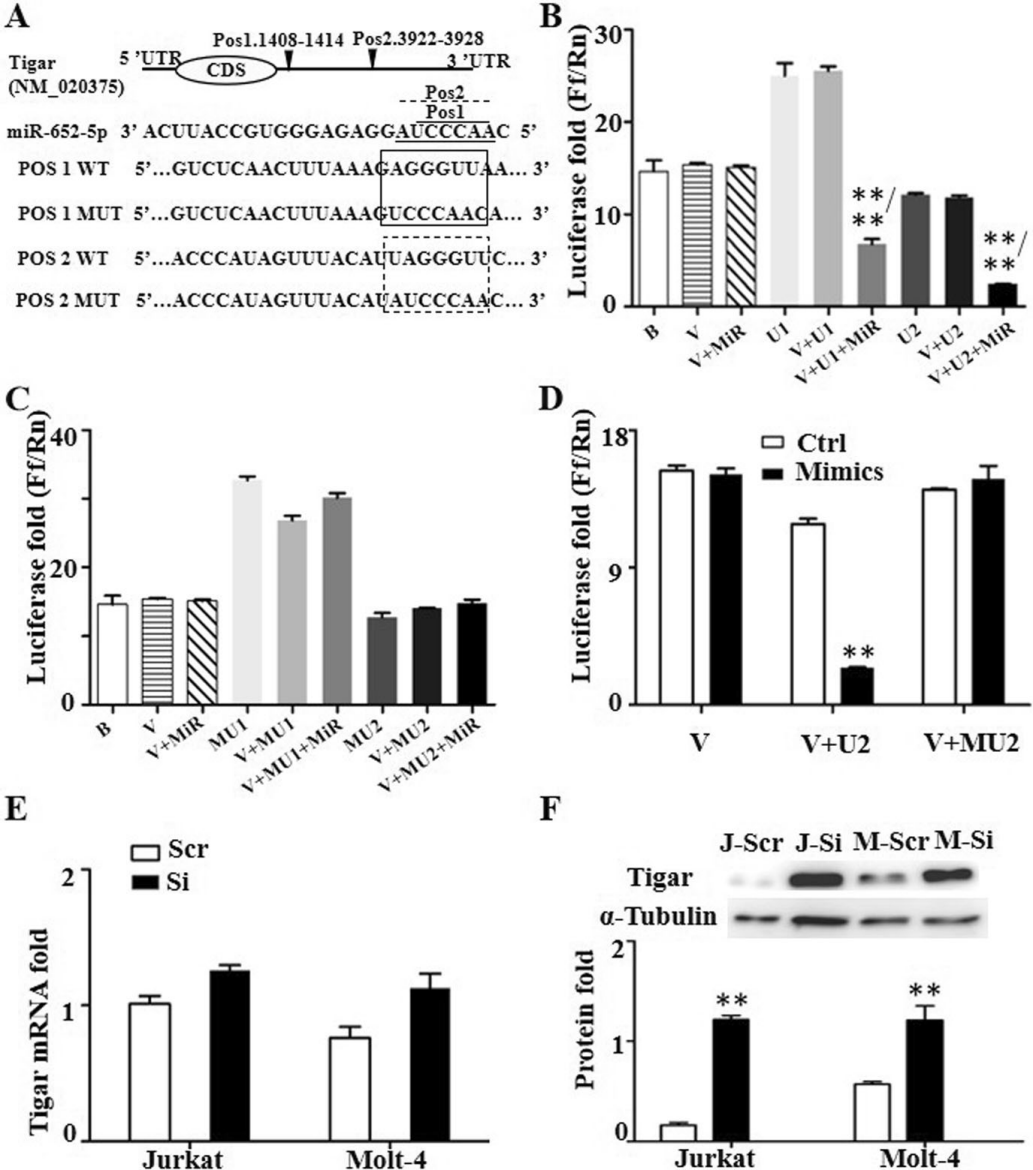
Inhibition of miR-652-5p on Tigar expression in T-ALL cells. (**A**) Conserved binding site for miR-652-5p within the Tigar mRNA 3ʹUTR as predicted by TargetScan software. Top, structural features of Tigar. The predicted binding site (bs) within the Tigar 3ʹUTR for miR-652-5p is indicated by inverted triangle. Middle, the sequence of miR-652-5p. Solid line indicated the first binding region, dotted line indicated the second binding region. Bottom, the sequence of fragment which is insert into plasmid for luciferase reporter assay. Relative luciferase activity in 293 T cells cotransfected with miR-652-5p and reporter or control luciferase plasmids containing the wildtype sequence of Tigar 3ʹUTR (**B**) or containing the mutated sequence (**C**). (**D**) Relative luciferase activity in 293 T cells cotransfected with miR-652-5p mimics and different reporter plasmid. The expression of Tigar mRNA (**E**) and protein (**F**) in Jurkat and Molt-4 cells with impaired miR-652-5p. Top indicated the representative image of Western Blot. Bottom indicated the statistics and normalized to control as 1.0 in F. Pos1., the binding position 1 for miR-652-5p to Tigar mRNA; Pos2., the binding position 2 for miR-652-5p to Tigar mRNA WT, the reporter plasmid with wildtype sequence of Tigar mRNA; MUT, the reporter plasmid with mutated sequence of Tigar mRNA; B blank; V, MiR vector with miR-652-5p; U1, vector with the first binding sequence for miR-652-5p to Tigar mRNA; U2, vector with the second binding sequence for miR-652-5p to Tigar mRNA; MU1, the mutated U1; MU2, the mutated U2; Scram, control; Si, impaired miR-652-5p; J, Jurkat cell lines as control; M, Molt-4 urkat cell lines. *p* is compared to control and calculated by *t* student; *p < 0.05; **p < 0.01. **/** indicated that the group was compared to V + MIR group/to V + U1or U2 group.

To verify the target gene of miR-652-5p, except for PFKFB2 and TIGAR, we detected also the expression of genes related with hematopoiesis (Prox1 and Pten), epigenetic modification (OTUD7B) and so forth, by Q-PCR (Supplementary Fig. 3C). The significantly difference were found in PFKFB2, TIGAR and OTUD7B mRNA level. Then, impaired miR-652-5p expression was found to enhance the protein expression of TIGAR significantly (Fig. 3F) and PFKFB2 protein slightly (Supplementary Fig. 3B). These results indicated that TIGAR is one of genes regulated by miR-652-5p.

The putative miR-652-5p target site in the 3ʹ-untranslated region (3ʹ-UTR) of TIGAR contains two putative 7-nucleotide regions (pos.1 1408 bp–1414 bp, pos.2 3922 bp–392 8 bp), matching with the seed region at the 5ʹ end of mature miR-652-5p, which was considered to be required for target recognition (Fig. 3A). We transiently cotransfected 293 T cell lines with a luciferase reporter plasmid containing wildtype or mutated TIGAR 3ʹ-UTR and miR-652-5p. The results showed that miR-652-5p inhibited about 80% of the 3ʹ-UTR luciferase reporter activity of TIGAR (Fig. 3B), but without effects on the luciferase activity of the reporter, in which the miR-652-5p-binding sites were mutated (Fig. 3C). We transiently transfected miR-652-5p mimics or the mimic control into 293 T cell lines; decreased luciferase reporter activity was observed only in the control group (Fig. 3D). The elevated TIGAR protein level was observed in Jurkat and Molt-4 cells transfected with impaired miR-652-5p (approximately six fold and two fold, respectively) compared with the controls (Fig. 3F), the increased level was found in the mRNA level (Fig. 3E). The results indicated that TIGAR was a direct target of miR-652-5p, and the repressed TIGAR by miR-652-5p predominantly attributed to posttranscriptional inhibition in T-ALL cells.

**Fig. 4:**
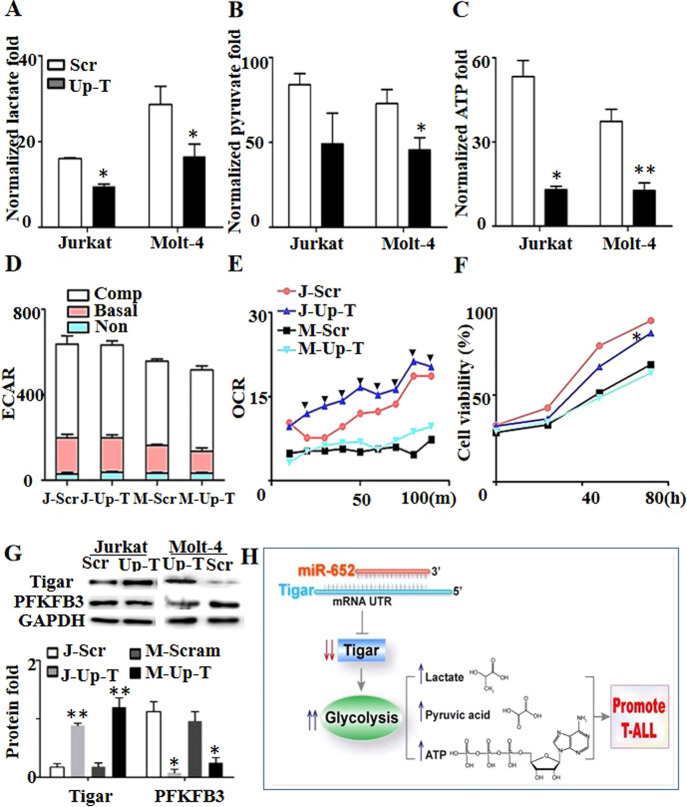
The role of over-expression of Tigar in T-ALL cells. Gas Chromatography-Tandem Mass detected (**A**) the lactate content, (**B**) pyruvate content, and the value was normalized to internal control. (**C**) Effect of over-expressed Tigar on the production of ATP. (**D**) Extracellular acidification rate (ECAR) and (**E**) Oxygen consumption rate (OCR) were detected by fluorescence microplate analyzer. ECAR was divided into three parts: non-glycolytic ECAR, basal ECAR (glycolytic ECAR) and compensatory ECAR with the stimulation by oligo. (**F**) Role of over-expressed Tigar on growth by CCK-8 assay. (**G**) The expression of protein. Top, the representative image of Western Blot. Bottom, the statistics and normalized to control as 1.0. (**H**) Schematic diaphragm showing the miR-652-5p-mediated pathway. Scr control, Up-T over-expressed Tigar, ECAR extracellular acidification rate, OCR oxygen consumption rate, m minutes, h hours, J Jurkat cell lines, M Molt-4 cell lines. Basal, basal glycosis; Non, the ECAR from non-glycosis; Comp, compensatory glycosis from stimulation with oligo. *p* is compared to control and calculated by *t* student; *p < 0.05; **p < 0.01., indicated the significance at certain time point, compared to control, *p* < 0.05.

Of note, TIGAR plays a dual role in cancer cell survival through regulating apoptosis and autophagy, as reported by Xie [21]. We constructed stable cell lines with overexpressed TIGAR (named J-Up-T and M-Up-T, respectively) to uncover the role of TIGAR in T-ALL (Fig. 4G). Overexpressed TIGAR in T-ALL cells decreased lactate production (Fig. 4A), pyruvate production (Fig. 4B), ATP production (Fig. 4C), and ECAR (Fig. 4D), and increased OCR (Fig. 4E), which were consistent with the effect of impaired miR-652-5p expression. However, the difference was not observed in the cell growth with overexpressed TIGAR, compared with control (Fig. 4F). These results indicated that glycolysis of T-ALL was regulated by miR-652-5p/TIGAR axis.

**Fig. 5:**
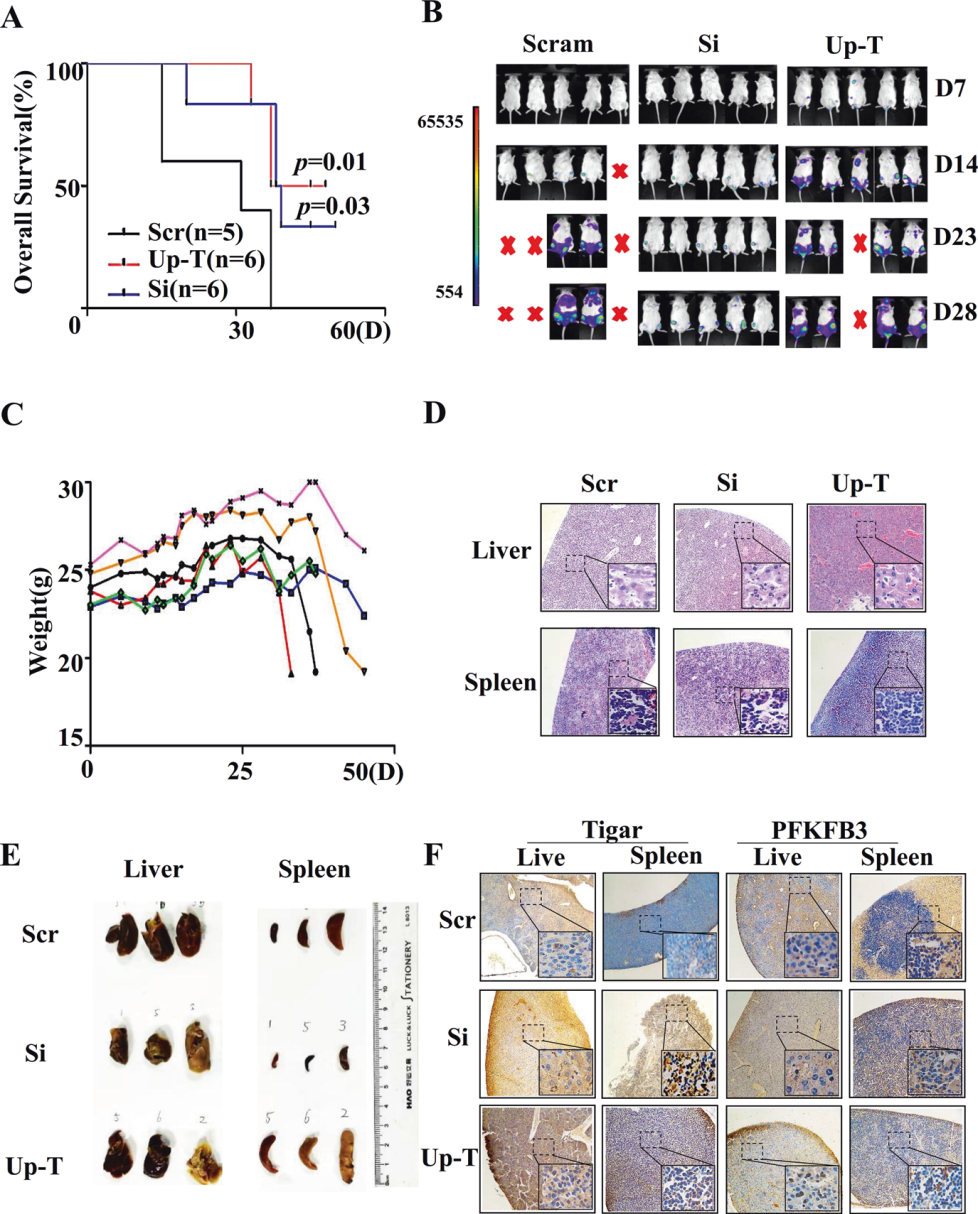
The role of miR-652-5p on T-ALL in vivo. Jurkat cell lines(Scr), Jurkat cell lines with impaired miR-652-5p(J-Si), and Jurkat with with over-expressed Tigar(J-Up-T) marked as GFP- and firefly luciferase-tagged were injected into NOD/SCID mice via the tail vein. (**A**) The overall survival. (**B**) GFP- and firely luciferase-tagged Jurkat cells with impaired miR-652-5p or control, were tail-vein injected into irradiated NOD/SCID mice (*n* = 5 per group). Bioluminescence images were obtained after administrating dynamically the mice D-Luciferin sodium salt. (**C**) The change of weight in group with impaired miR-652-5p. (**D**) HE staining in sections from liver and spleen tissue. (**E**) The tissue of liver and spleen. (**F**) Immunostaining with anti-Tigar antibody or anti-PFKFB3 antibody. Scr, control; Si, impaired miR-652-5p; Up-T, over-expressed Tigar; D, days. *p* is compared to control and calculated by Log-rank.*p < 0.05; **p < 0.01.

Finally, the relevance of these findings in vivo was investigated. The mice were randomized into three groups with different treatments, including Jurkat-injected, J-Si-injected, and J-Up-T-injected groups. Importantly, the median survival time was 31 days, 43.5 days, and 38.5 days in the Jurkat-injected, J-Si-injected, and J-Up-T injected groups, respectively (Fig. 5A). This finding indicated that both impaired miR-652-5p and overexpressed TIGAR prolonged the OS of mice with leukemia, and the OS was extended more in the J-Si-injected group than in the J-Up-T-injected group. In the mice with J-Si, increase in weight was the least (Fig. 5C and Supplementary Fig. 5A and 5B), the Jurkat cell line engraftment was delayed obviously by bioluminescence imaging (Fig. 5B), the spleen tissue was smaller(Fig. 5E) and the degree of infiltration of was decreased in spleen and liver tissue by HE staining(Fig. 5D). Additionally, the expression of PFKFB3 and TIGAR proteins was detected by immunohistochemistry. The PFKFB3 expression increased in leukemic mice with impaired miR-652-5p, and reduced in leukemic mice with overexpressed TIGAR (Fig. 5F). Therefore, we concluded that impaired miR-652-5p elevated the expression of TIGAR, thus repressing glycolysis and prolonging the OS of T-ALL.

## Discussion

Increased miR-652-5p expression was found in T-ALL with impaired ARRB1. However, we demonstrated that independent impaired miR-652-5p could inhibit proliferation, promote apoptosis in vitro, and prolong the OS in vivo. Further, we uncovered that decreased miR-652-5p expression suppressed the glycolysis level in T-ALL by targeting the 3ʹ-UTR of TIGAR mRNA. These data shed light on a previously unexpected role of miR-652-5p in T-ALL and showed the effect of glycolysis on T-ALL via the miR-652-5p/TIGAR axis.

The expression of miR-652-5p is increased significantly in 5/13 patients(Supplementary Figure 1C), the 5 patients were divided into different subtype including: 2 cortical-T-ALL, 2 pre-T-ALL and 1 pro-T-ALL. And the postinduction minimal residual disease(MRD) at 19 days is 0.01%, <0.01%, 0.71%, 7.31% and 38.79%, respectively (Supplementary Table 1). According to the results by Boissel N [22] and Radich JP [23], MRD is a predictor for the prognosis of leukemia. We speculated that miR-652-5p might indicate the unfavorable prognosis for the patients with pediatric T-ALL. More patients need to be analyzed for demonstrating the speculation.

miRs, as vital factors, play a role in cell differentiation, proliferation, plasticity, metabolism, and other cell founctions [24, 25]. In this study, the proliferation, apoptosis, ROS level, and cell cycle were observed in vitro and metastasis in vivo. Impaired miR-652-5p promoted apoptosis and reduced metastasis significantly in T-ALL, which was contradictory to the role of miR-652-5p in esophageal squamous cell carcinoma [26]. The dual functional role and tissue specificity of miRs in cancer progression [27, 28] might explain this contradiction, which also was one of the reasons for diverse roles of miR-652-5p in subtypes of T-ALL, targeting multiple genes and complex functions.

The roles of miRs depend on the target genes to work. GO enrichment for the target genes showed that miR-652-5p might affect the development in the ventricular system, pancreas, lung, and spermatids. Also, miR-652-5p might be involved in cancer progression related to critical signaling pathways such as Wnt, glucose metabolism, and so forth. We explored that miR-652-5p targeted P62, thus regulating the intracellular ROS content in T-ALL (accepted by Zhongguo Shi Yan Xue Ye Xue Za Zhi on May 2021). In fact, the change in total ROS content by impaired miR-652-5p was not much (Fig. 1C). Hence, glucose metabolism in patients with a higher enrichment score was explored in the study. Besides TIGAR and PFKFB2 proteins related to glycolysis, the expression of OTUD7B, PROX1, and PTEN proteins was also increased by impaired miR-652-5p. Considering multiple targets for a given miR, the possibility that the genes targeted by miR-652-5p contributed to abnormal hematopoiesis or glycolysis or cell growth was not eliminated.

Notably, the relationship of glycolysis with cell growth and apoptosis has been claimed massively. Lactate dehydrogenase inhibitors resulted in reduced glycolysis and tumor growth [29]. HSP90 promoted cell glycolysis and proliferation and inhibited apoptosis [30]. How glycolysis affected the growth or apoptosis needed further exploration. Peng et al. demonstrated that activated glycolysis promoted stem-like traits of breast cancer cells under hypoxia [31]. Zhang T indicated that glycolysis provided energy for the survival and proliferation of cancer cells [32]. In the present study, the ATP level was decreased by impaired miR-652-5p (Fig. 2C) and overexpressed TIGAR (Fig. 4C). We speculated that the decrease in the ATP level might be the cause for the slow growth of T-ALL cells, but more evidence is needed to support the idea.

TIGAR is one of the target genes for miR-652-5p, an endogenous inhibitor of glycolysis [33]. Previous literature reported that TIGAR retuned cell metabolism from glycolysis, lowered intracellular ROS levels, suppressed autophagy in response to nutrient starvation or metabolic stress, and functioned to inhibit apoptosis [34]. More interestingly, we found that impaired miR-652-5p inhibited glycolysis but induced apoptosis in T-ALL. In fact, either a positive [35] or a negative [36–38] relationship between glycolysis and apoptosis existed in diseases. Hence, we inferred that apoptosis was due to other target genes of miR-652-5p, or glycolysis and apoptosis were co-regulated by other genes in T-ALL.

Both TIGAR and PFKFB2 were enrichments in the fructose 2,6-bisphosphate metabolic process (GO: 0006003). PFKFB2 is a member of the PFKFB (6-phosphofructo-2-kinase /fructose-2,6- bisphosphatases) family and increases the glycolysis level [39]. Our results showed that the TIGAR level was increased significantly, and the PFKFB2 level was increased slightly by impaired miR-652-5p, indicating that the pathways related to increasing or decreasing glycolysis occurred simultaneously. Meanwhile, PFKFB3 had the highest kinase activity in the PFKFB kinase family [39]. Simon-Molas et al. demonstrated crosstalk between PFKFB3 and Tigar [40]. We demonstrated that increased TIGAR level inhibited the expression of PFKFB3 protein in vitro (Fig. 4G) and in vivo (Fig. 5F), implying that decreased glycolysis was mainly due to reduced PFKFB3 in T-ALL with impaired miR-652-5p, and overexpressed TIGAR would repress glycolysis via the crosstalk between TIGAR and PFKFB3.

We observed that both impaired miR-652-5p and overexpressed TIGAR slowed down the growth rate and prolonged the OS. However, the effect of impaired miR-652-5p was more remarkable. The dual role of TIGAR might be one of the reasons for this, besides the involvement of other possible target genes of miR-652-5p in the process (ETS1 [41] and HIF1α [42]), suggesting that miR-652-5p might be a more suitable target in therapeutic intervention.

In summary, our results indicated that miR-652-5p/TIGAR induced glycolysis by regulating PFKFB3 expression, eventually contributing to decreased apoptosis in vitro and shortened survival time in vivo, which provided a basis for targeted miR-652-5p to repress the glycolysis in T-ALL. Future studies on miR-652-5p might provide further insights into prognosis evaluation and treatment strategies for T-ALL.

## Supplementary information

supplmentary

checklist

## Data Availability

All data generated or analyzed during this study are included in this published article [and its supplementary information files]
