# Supplementary material for: MiR-652-5p elevated glycolysis level by targeting TIGAR in T-cell acute lymphoblastic leukemia
Source: Cell Death Dis. 2022 Feb 14;13(2):148. doi: 10.1038/s41419-022-04600-7 (PMC8844069; doi:10.1038/s41419-022-04600-7)
Supplement: Supplementary file 1 — supplmentary [file 41419_2022_4600_MOESM1_ESM.doc]

| **No.** | **Gender** | **Age**  **(Y)** | **WBC (10^9^/L)** | **Blast cell**  **(%)** | **Immunologic feature** | **Cytogenetic marker** | **Molecular marker** | **MRD**  **(%)** |
| --- | --- | --- | --- | --- | --- | --- | --- | --- |
| P1 | M | 5 | 744.24 | 79.5 | cortical-T-ALL | 46, XY[20] | / | / |
| P2 | F | 1 | 534.79 | 99.1 | cortical-T-ALL | 46, XX(3) | / | 0.01% |
| P3 | M | 14 | 46.43 | 87.1 | medullary-T-ALL | 46, XY[12] | SIL/TAL1(+) | (-) |
| P4 | M | 4 | 123.06 | 93.0 | cortical-T-ALL | 46,XY,DEL(6)(q21)[3]/46,xy[5] | / | 6.92% |
| P5 | M | 14 | 106.67 | 92.3 | cortical-T-ALL | 46, XY[6] | / | <0.01% |
| P6 | M | 10 | 239.55 | 97.6 | pre-T-ALL | 46, XY[20] | / | <0.01% |
| P7 | M | 9 | 26.53 | 98.1 | pre-T-ALL /cortical-T-ALL | / | / | 0.71% |
| P8 | M | 7 | 483.15 | 91.6 | pre-T-ALL | 46, XY[1] | SIL/TAL1(+) | 7.31% |
| P9 | M | 12 | 48.17 | 80.0 | cortical-T-ALL | 46, XY[20] | SIL/TAL1(+) | <0.01% |
| P10 | M | 14 | 17.18 | 75.8 | pro-T-ALL | 46, XY[2] | MLL-ENL(+), HOX11(+) | 38.79% |
| P11 | M | 9 | 404.33 | 95.2 | medullary-T-ALL | 46, XY[4] | SIL/TAL1(+) | 0.57% |
| P12 | M | 8 | 38.1 | 93.2 | cortical-T-ALL | / | / | / |
| P13 | M | 8 | / | / | cortical-T-ALL | / | / | / |
| C1 | M | 8 | 4.81 | NA | | | | |
| C2 | M | 13 | 6.23 | NA | | | | |
| C3 | F | 5 | 4.38 | NA | | | | |
| C4 | M | 10 | 4.69 | NA | | | | |
| C5 | M | 5 | 5.12 | NA | | | | |

**Suppl Table 1. The clinical characters for patients and controls**

**Abbreviation:** No., number; P, patient; C, control; WBC, leukocyte count in peripheral blood; MRD, minimal residual disease on 19th day after induction chemotherapy; M, male; F, Female; Y, year.

**Suppl Table 2. The sequence of primers and insert fragments**

| **Purpose** | **Name (vector)** | **Sequence** |
| --- | --- | --- |
| **Plasmids** | **Si-miR-652-5p**  **(pSEBR-CIR)** | F: AGCTTTGAATGGCACGGAGTCCTAGGGTTGACGGTATCTGAATGG  CACGGAGTCCTAGGGTTGG  R: GATCCCAACCCTAGGACTCCGTGCCATTCAGATACCGTCAACCCT  AGGACTCCGTGCCATTCAA |
|  | **Up-Tigar (pSEH-361)** | F: TATCTTGTGGAAAGAGATCTCTC  R: CGCCTCCGGGCCCTAGGGATAAC |
|  | **Tigar U1**  **(PmirGLO)** | GATAACCACAGCTGTGGTTATTTTGTGGCAGGTACAGGTGAGGTTGCCTCTTCTTTCCACCAGATGGCACTGGAGAAAAGGGATTTTGATTAACTTAATTTTATACACAAGATTGGTCTTTCAGAAATCTTTATCTAAATGAAGAAACTGTTCTACATGTAGTTCGCTTTTAAGTCACCATGTTGCTAAATGCTGTGAGTCTCAACTTTAAAGAGGGTTAATACTAAAGTTGTCAGTTAAGGCTATAGTCACATATAGTCAAAATTTACTTTGACATCATTTTAAATCATATGTAAAATTCAGTATTATATTGTTCTTAGTGTGTCTAGCACTACCAGTTTTCTACCAGTATTGAAACAGGGCACGTGTTTGAGCATCAGAGGAAGTATACTGTAAATAAAAATTTGAAAAATAAGC |
|  | **Tigar U2**  **(PmirGLO)** | AATGAATTTTTTAAGAGACTTAATTTTTTAAGAGAAACTTTAATTTCCCAGCAAAAGTGAGAGGAAGGTAGAGAGATTTCCCATCTATCCCCTGCCCCTACACACGCATTGCTTCTCCTACTATCAACATCCCCCACCAGACCAGTGCATCTGTTACCATCGGTGAGCCCACATTACACTGACATATCCACATCACCTGAA**ACCC**ATAGTTTACATTAGGGTTCATTCTTGGTGTTGCATGATCTGTGGGTTTGGACAAACGTATGAAGACATGTATTCCACCATTATAGTATCATGCAGAGTATTTTCACTGCCCTAAAAGTCCTCTGTGTTCCCTGTACTCATCTTTCCCACCTTGTCTTGCCTGTTTCTGTTAGGTTTAGTCCGAGGCCTAGCAGAGGGAATGGGCAGAAGTTTCCAGTTCCAG |
|  | **Tigar MUT1**  **(PmirGLO)** | GATAACCACAGCTGTGGTTATTTTGTGGCAGGTACAGGTGAGGTTGCCTCTTCTTTCCACCAGATGGCACTGGAGAAAAGGGATTTTGATTAACTTAATTTTATACACAAGATTGGTCTTTCAGAAATCTTTATCTAAATGAAGAAACTGTTCTACATGTAGTTCGCTTTTAAGTCACCATGTTGCTAAATGCTGTGAGTCTCAACTTTAAAGTCCCAATATACTAAAGTTGTCAGTTAAGGCTATAGTCACATATAGTCAAAATTTACTTTGACATCATTTTAAATCATATGTAAAATTCAGTATTATATTGTTCTTAGTGTGTCTAGCACTACCAGTTTTCTACCAGTATTGAAACAGGGCACGTGTTTGAGCATCAGAGGAAGTATACTGTAAATAAAAATTTGAAAAATAAGC |
|  | **Tigar MUT2**  **(PmirGLO)** | AATGAATTTTTTAAGAGACTTAATTTTTTAAGAGAAACTTTAATTTCCCAGCAAAAGTGAGAGGAAGGTAGAGAGATTTCCCATCTATCCCCTGCCCCTACACACGCATTGCTTCTCCTACTATCAACATCCCCCACCAGACCAGTGCATCTGTTACCATCGGTGAGCCCACATTACACTGACATATCCACATCACCTGAAACCCATAGTTTACATATCCCAACATTCTTGGTGTTGCATGATCTGTGGGTTTGGACAAACGTATGAAGACATGTATTCCACCATTATAGTATCATGCAGAGTATTTTCACTGCCCTAAAAGTCCTCTGTGTTCCCTGTACTCATCTTTCCCACCTTGTCTTGCCTGTTTCTGTTAGGTTTAGTCCGAGGCCTAGCAGAGGGAATGGGCAGAAGTTTCCAGTTCCAG |
| **Q-PCR** | **GAPDH** | F: CAGCGACACCCACTCCTCCACCTT  R: CATGAGGTCCACCACCCTGTTG |
|  | **TIGAR** | F: CCAAAGCAGCCAGGGAAGAGTG  R: CCGCTTCTTTCAGGATTAGTTGAC |
|  | **miR-652-5p** | R: CTCAACTGGTGTCGTGGAGTCGGCAATTCAGTTGAGTGAATGGC  F: ACACTCCAGCTGGGCAACCCTAGGAGAGGGTGC;  R: CTCAACTGGTGTCGTGGA |
|  | **HK** | F: TGCCAACATTCGTAAGGTCCA  R: TGTCATAAACCTCGGACTCCA |
|  | **GLUT1** | F: CGGGCCAAGAGTGTGCTAAA  R: TGACGATACCGGAGCCAATG |
|  | **LDHA** | F: ATCTTGACCTACGTGGCTTGGA  R: CCATACAGGCACACTGGAATCTC |

**Abbreviation**: Si-miR-652-5p, impaired expression of miR-652-5p; Up-Tigar, over-expressed Tigar; F, forward primer; R, reverse primer; Q-PCR, quantitative polymerase chain reaction.

**
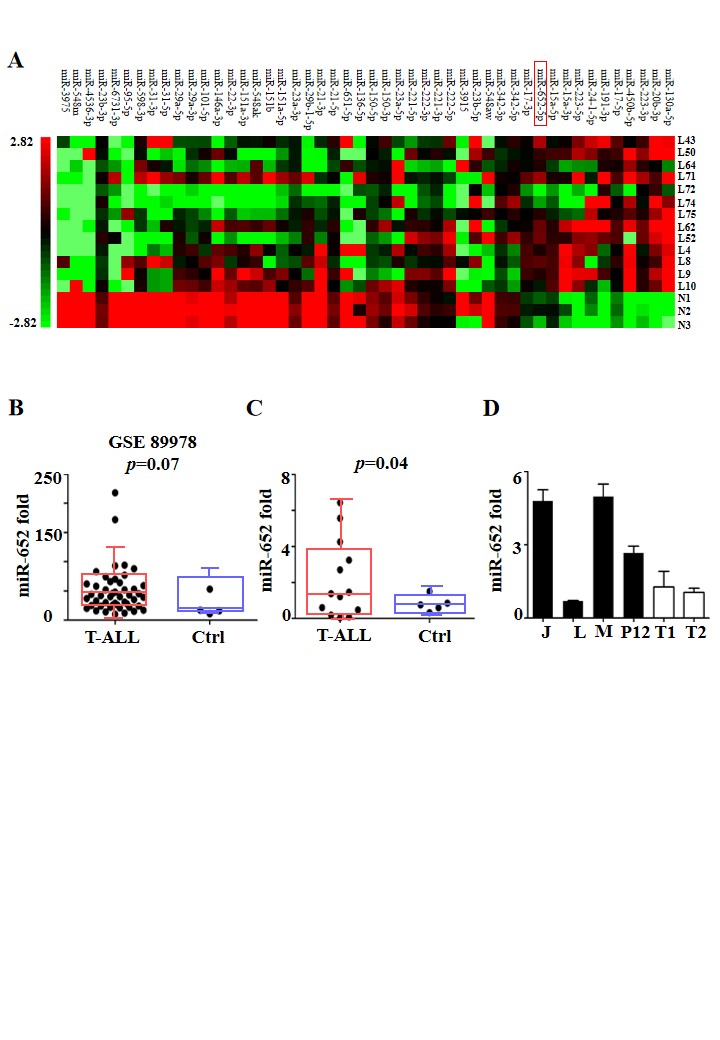
Suppl Figure 1. The expression of miR-652-5p in T-ALL**

The expression of miR-652-5p were analyzed in (**A**) RNA-Seq data and (**B**) GSE89978 data, with the filter average intensity >10, fold>2.0 or <0.5. The identify the expression of miR-652-5p by Q-PCR (**C**) in 13 bone marrow cells from pediatric T-ALL patients and 5 thymocytes from pediatric healthy donor; (**D**) in 4 T-ALL cell lines and 2 thymocytes from pediatric healthy donor.

*Abbreviation*: L43, L50….L9 indicate the number of T-ALL patients; N1….N3 indicates healthy donor; T1 and T2 indicate the thymocytes as control. Ctrl, control; J, Jurkat cell line; L, Loucy cell line; M, Molt-4 cell line; P12, P12 cell line; *p* is compared to control and calculated by *t* student.

**
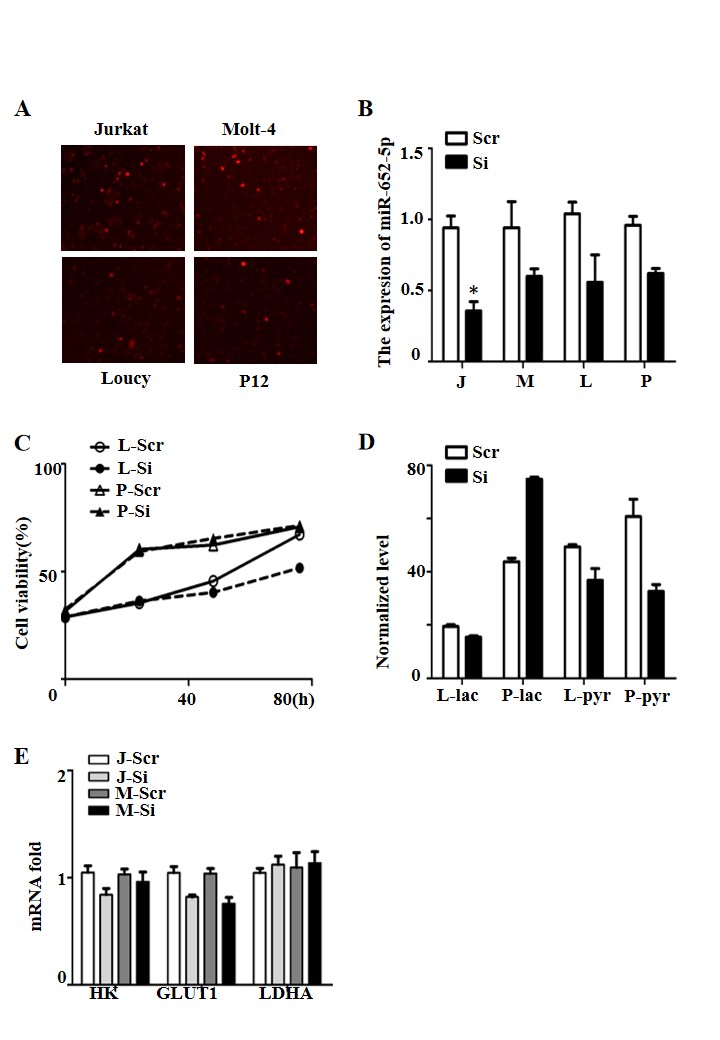
Suppl Figure 2.The effects of miR-652-5p on T-ALL cells**

(**A**) The representative image of stable cell lines with impaired miR-652-5p. (**B**)The identification expression of miR-652-5p by Q-PCR. (**C**) The growth of cell lines by CCK-8. (**D**) The lactate and pyruvate level by Gas Chromatography-Tandem Mass. (**E**). The expression of mRNA. Control as 1.0.

*Abbreviation*: h, hours. Scr, control; Si, impaired miR-652-5p; L, Loucy cell lines; P, P12 cell lines; J, Jurkat cell lines; M, Molt-4 cell lines; lac, Lactate; pyr, Pyruvate.

**
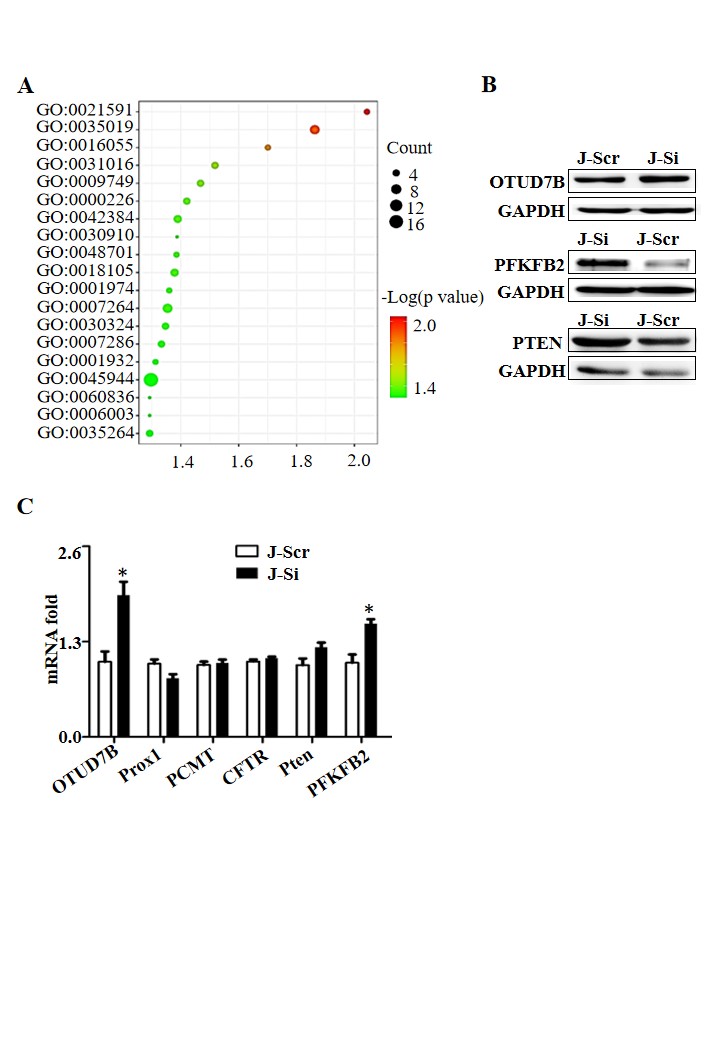
Suppl Figure 3. The target genes of miR-652-5p**

(**A**) Top 19 GO terms enriched by DAVID software, for 205 possible target genes of miR-652-5p. The 205 genes were derived from the intersection of target genes predicted by miRbase software, TargetScan 7.0 software respectively. (**B**) The target genes protein level of miR-652-5p. (**C**) The target genes mRNA level of miR-652-5p.

*Abbreviation*: J-Scr, Jurkat cell lines with empty plasmid; J-Si, Jurkat cell lines with impaired miR-652-5p.

*p* is compared to control and calculated by *t* student. **, indicated *p*<0.01.

**
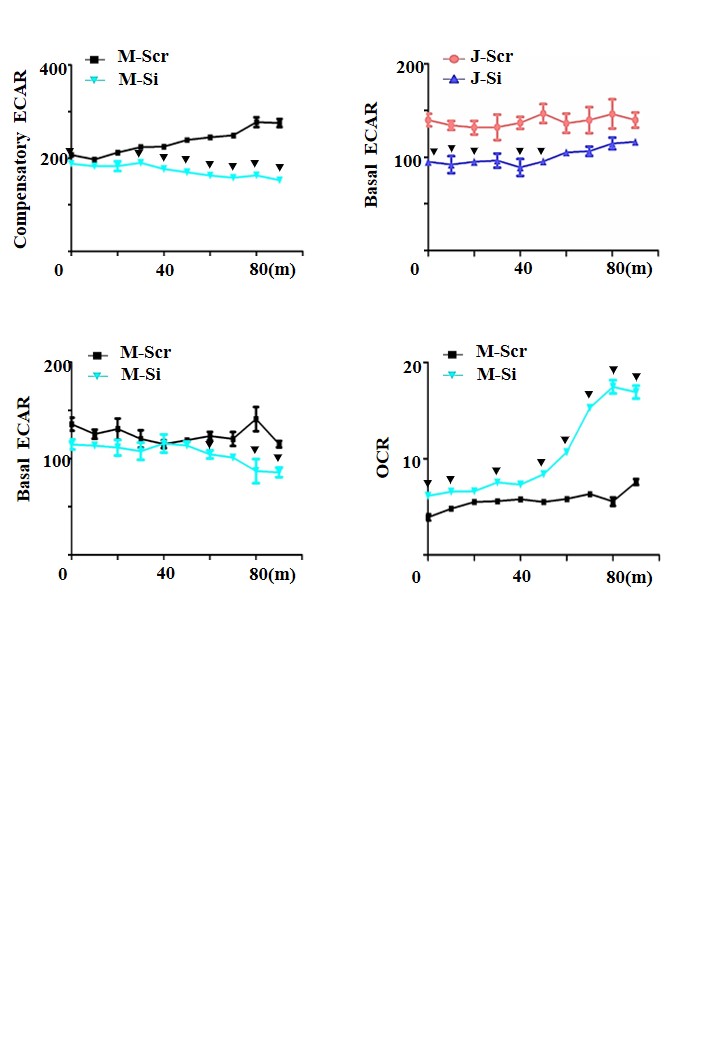
Suppl Figure 4. The time curve of extracellular acidification rate in different T-ALL cells**

(**A**) Continuous monitoring compensatory ECAR in Molt-4 cell line; Continuous monitoring basal ECAR in (**B**) Jurkat cell line and (**C**) Molt-4 cell line; (**D**) Continuous monitoring OCR in Molt-4 cell line.

*Abbreviation*: ECAR, Extracellular acidification rate; m, minutes; OCR, Oxygen consumption rate; Scr, control; Si, impaired miR-652-5p; J, Jurkat cell line; M, molt-4 cell line; m, minutes.

, indicated the significance at certain time point, compared to control, *p*<0.05.

**
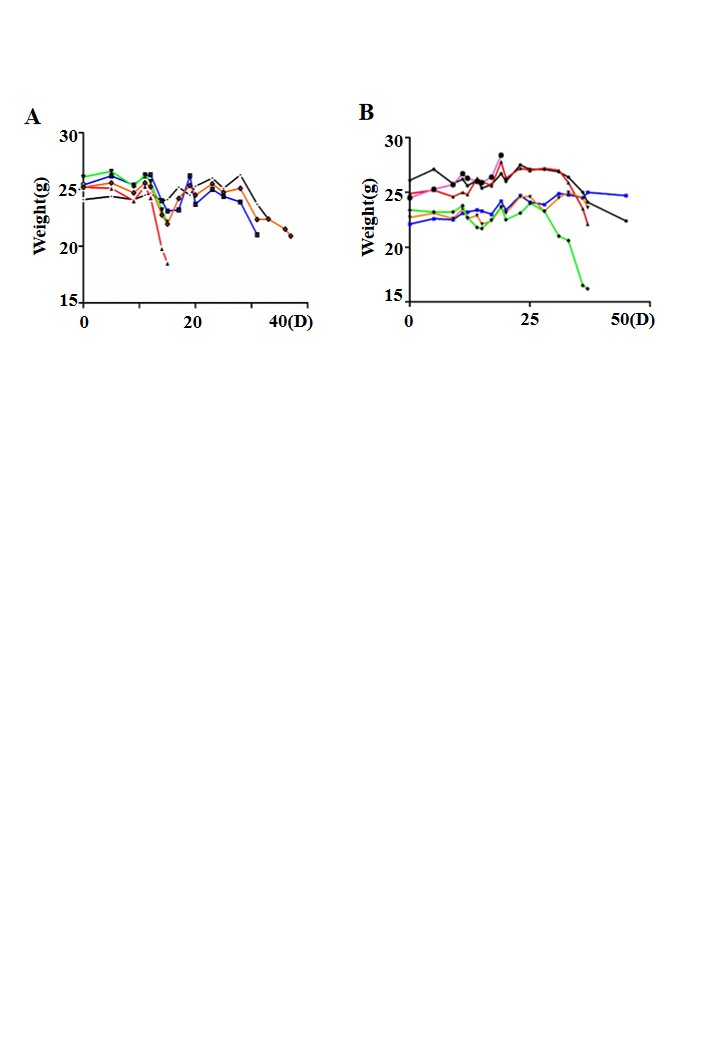
Suppl Figure 5. The reconstruction of leukemia in vivo**

The weight of every mouse in (**A**) control group and (**B**) over-expressed Tigar group.

*Abbreviation*: D, days.
